# A pathway analysis-based algorithm for calculating the participation degree of ncRNA in transcriptome

**DOI:** 10.1038/s41598-022-27178-8

**Published:** 2022-12-31

**Authors:** Xinyi Gu, Shen Wang, Bo Jin, Zhidan Qi, Jin Deng, Chen Huang, Xiaofeng Yin

**Affiliations:** 1 Department of Orthopedics and Traumatology, Peking University People’s Hospital, Beijing, 100044 China; 2 Key Laboratory of Trauma and Neural Regeneration (Peking University), Beijing, China

**Keywords:** Cell biology, Genetics

## Abstract

After sequencing, it is common to screen ncRNA according to expression differences. But this may lose a lot of valuable information and there is currently no indicator to characterize the regulatory function and participation degree of ncRNA on transcriptome. Based on existing pathway enrichment methods, we developed a new algorithm to calculating the participation degree of ncRNA in transcriptome (PDNT). Here we analyzed multiple data sets, and differentially expressed genes (DEGs) were used for pathway enrichment analysis. The PDNT algorithm was used to calculate the Contribution value (C value) of each ncRNA based on its target genes and the pathways they participates in. The results showed that compared with ncRNAs screened by log2 fold change (FC) and p-value, those screened by C value regulated more DEGs in IPA canonical pathways, and their target DEGs were more concentrated in the core region of the protein–protein interaction (PPI) network. The ranking of disease critical ncRNAs increased integrally after sorting with C value. Collectively, we found that the PDNT algorithm provides a measure from another view compared with the log2FC and p-value and it may provide more clues to effectively evaluate ncRNA.

## Introduction

One of the most important applications of RNA sequencing is to compare the differences in the expression of the non-coding RNAs (ncRNAs). ncRNA refers to a kind of RNA that can be transcribed from the genome but not translated into proteins and can perform their biological functions at the RNA level, including rRNA, tRNA, snRNA, lncRNA, microRNA (miRNA) and others. They play important roles in normal development, physiology and disease^1^. miRNA and lncRNA are ncRNAs that have been widely studied and have been confirmed to have the strong regulatory ability on gene expression^2–6^. By direct or indirect means, a single miRNA or lncRNA can regulate hundreds of mRNAs.

High throughput sequencing is a common method for ncRNA research. People often select genes with high expression differences for follow-up function research^9,10^. In the traditional way, using log2 FC and p-value as thresholds to screen ncRNAs will obviously lose a lot of valuable information. In order to screen ncRNAs more scientifically, many analysis methods have been derived. There are many enrichment analysis methods and databases, such as GSEA^11^ IPA^12^, David^13^, Catmap^14^ and GlobalTest^15^. Their analytical methods have different priorities, but the general idea is the same, that is, to perform functional annotation on the RNA profile. But through these methods, we can only observe which genes and pathways are associated with ncRNAs. We do not have an indicator to measure the the regulatory function and participation degree of ncRNA on transcriptome expression. This lack will cause us to miss a lot of valuable information when we screen ncRNAs. Here, we developed an algorithm PDNT, through which we can get the contribution value (C value) of each ncRNA. C value is defined as a quantitative indicator of the participation degree of ncRNA in transcriptome. The algorithm is, (1) Enrich the pathways with DEGs in the dataset, and then use the −lg (p-value) of these pathways as the weighted phase; (2) Take the intersection of the target gene of ncRNA and DEGs, and calculate the proportion of this intersection in each pathway; (3) C value is equal to the weighted sum of these proportions. To verify the utility of the C value, we collected the existing sequencing results, including skeletal muscle denervation, Alzheimer's disease, prostate cancer, gastric cancer, and adipocyte differentiation. C57BL/6 mice were used as the model of skeletal muscle denervation, APP/PS1 mice as the model of Alzheimer's disease, prostate cancer, gastric cancer, and adipocyte differentiation samples were all from human^16–20^.

Our proposed algorithm PDNT takes into accounts the p-value for each enriched pathway and the proportion of ncRNA target genes in each pathway. We expect to quantify the participation degree of ncRNA in transcriptome, and to optimize the efficiency of screening ncRNA after high throughput sequencing.

## Results

### The C value of each DE ncRNA is equal to the sum of BP value, CC value, MF value and KEGG value

We calculated the C value of each DE miRNA in skeletal muscle denervation, prostate cancer, Alzheimer's disease and gastric cancer data sets respectively. In addition, we calculated the C value of each lncRNA in skeletal muscle denervation and adipocyte differentiation data sets. The details of these data were aggregated into a table (Table 1). The C values of each DE ncRNA based on biological process (BP), cellular component (CC), molecular function (MF) and KEGG analysis can be obtained, and we named these C values as BP value, CC value, MF value and KEGG value respectively. The total C value of each DE ncRNA was equal to the sum of BP value, CC value, MF value and KEGG value. The DE miRNAs were sorted with the total C value to obtain the 10 DE miRNAs with maximum C value, named as top10 C value miRNAs (Table 2). The top10 DE miRNAs with maximum absolute Log2 FC (top10 FC miRNAs), and the top10 DE miRNAs with minimum p-value (top10 p-value miRNAs), were obtained by sorting the DE miRNAs according to the absolute Log2 fold FC and p-value respectively (Supplementary Tables 1, 2). Similarly, DE lncRNAs were processed in the same way to obtain top5 C value lncRNAs, top5 FC lncRNAs, top5 p-value lncRNAs for adipocyte differentiation and top10 C value lncRNAs, top10 FC lncRNAs, top10 p-value lncRNAs for skeletal muscle denervation (Table 3, Supplementary Tables 3–6).

**Table 1:** Description of publicly available data sets used in the meta-analysis.

RNA	Gene expression platforms	Status	Tissue	Organism	Ref
MicroRNA	Illumina HiSeq X	Nerve resection	Muscle	Mus Musculus	^16^
MicroRNA	Illumina HiSeq 2500	Alzheimer’s disease	Brain	Mus Musculus	GSE132177^17^
MicroRNA	Agilent-019118	Prostate cancer	Tumor tissue	Homo sapiens	GSE64318^21^
MicroRNA	Illumina HiSeq 2000 miRNAseq	Gastric cancer	Tumor tissue	Homo sapiens	^19^
LncRNA	Illumina HiSeq X	Nerve resection	Muscle	Mus Musculus	^16^
LncRNA	Illumina HiSeq 1500	Adipocyte differentiated stem cell	Adipose-derived stem cell	Homo sapiens	GSE113253^20^

Ref: reference.

**Table 2:** The top10 miRNAs according to C value.

miRNAs	KEGG value		BP value	CC value		MF value	C value
**Skeletal muscle denervation**							
mmu-miR-1943-5p	33.2298		816.2096	57.7971		86.4830	993.7195
mmu-miR-322-5p	30.8406		752.9168	68.5535		79.5975	931.9084
mmu-miR-497a-5p	30.7342		748.7866	69.7075		79.4659	928.6942
mmu-miR-674-5p	27.1606		715.4414	58.0113		72.7104	873.3236
mmu-miR-377-3p	27.4901		693.5040	53.2729		72.8327	847.0997
mmu-miR-378d	23.2596		680.9893	61.2806		72.2897	837.8192
mmu-miR-486a-3p	26.8248		657.0155	50.6866		69.4835	804.0103
mmu-miR-34a-5p	26.6988		659.2445	53.4273		63.0869	802.4575
mmu-miR-34c-5p	26.6988		659.2445	53.4273		63.0869	802.4575
mmu-miR-485-5p	24.7998		631.6504	56.8839		69.4729	782.8069
**Alzheimer’s disease**							
mmu-miR-340-5p	43.5208	1010.0391			99.4072	95.1248	1248.0919
mmu-miR-128-3p	32.3406	702.2785			72.0400	72.0975	878.7565
mmu-miR-1912-3p	31.4818	665.3036			71.0238	65.4024	833.2115
mmu-miR-3065-5p	28.5725	635.0081			59.7389	60.1966	783.5160
mmu-miR-30e-5p	25.0791	603.9772			61.3365	55.3197	745.7125
mmu-miR-30b-5p	24.3197	578.0747			60.5463	54.1156	717.0563
mmu-miR-369-3p	21.9838	578.5994			50.7141	53.2306	704.5279
mmu-miR-30f.	23.9495	503.5940			55.6817	48.9650	632.1902
mmu-miR-16-5p	24.3638	493.9211			47.4183	46.5204	612.2236
mmu-miR-3470a	18.4495	405.6942			42.6480	40.2364	507.0280
**Prostate cancer**							
hsa-miR-374a-5p	4.3985	118.2693			5.2133	10.0440	137.9250
hsa-miR-513a-5p	5.6572	112.0295			6.9103	12.5930	137.1900
hsa-miR-95-5p	3.4669	116.9228			5.4779	9.5689	135.4365
hsa-miR-374b-5p	3.8076	113.5990			5.3002	11.6734	134.3802
hsa-miR-498	4.7281	107.2751			5.8249	10.8348	128.6630
hsa-miR-20a-5p	4.1156	109.1116			5.6328	8.0808	126.9408
hsa-miR-30e-5p	3.6117	102.8695			5.2738	8.0756	119.8306
hsa-miR-96-5p	3.0537	94.3429			5.1002	6.5807	109.0776
hsa-miR-148a-5p	3.3918	90.1048			4.5191	6.8996	104.9153
hsa-miR-429	3.2433	85.7535			5.0370	7.6430	101.6768
**Gastric cancer**							
hsa-miR-153-5p	18.2391	362.6950			64.0236	71.7660	516.7236
hsa-miR-3662	15.3946	317.1733			49.4005	52.8578	434.8263
hsa-miR-548f.-3p	14.2178	286.8668			49.4087	47.7987	398.2921
hsa-miR-5680	13.2793	242.4315			42.3071	49.8912	347.9091
hsa-miR-944	14.7858	239.0951			40.7592	49.7775	344.4176
hsa-miR-7–2-3p	13.3438	249.0496			38.5958	38.1965	339.1857
hsa-miR-4677-5p	8.4194	187.5756			34.2620	30.5864	260.8433
hsa-miR-20a-5p	7.5578	178.5451			36.1427	28.2557	250.5012
hsa-miR-4728-5p	10.0061	161.1989			32.7904	31.1540	235.1493
hsa-miR-6507-5p	10.3778	162.0089			28.4527	26.2513	227.0907

*BP*, biological process; *CC*, cellular component; *MF*, molecular function.

**Table 3:** The top lncRNAs according to C value.

miRNAs	KEGG value	BP value	CC value	MF value	C value
**Adipocyte differentiation**					
MIAT	0.7422	33.6464	1.7731	4.2158	40.3777
CYTOR	0.5408	27.4816	1.6461	3.5501	33.2186
LINC02202	0.8555	23.7874	1.6544	2.7816	29.0790
OSER1-DT	0.7450	22.7320	1.7143	2.1406	27.3320
LINC01119	0.3521	21.7651	1.1976	2.0853	25.4002
**Skeletal muscle denervation**					
LNC_000596	19.4847	283.7968	35.8261	40.4197	379.5275
ENSMUST00000138653.7	18.6985	268.8787	31.0684	39.4189	358.0646
ENSMUST00000131642.1	17.0027	256.5569	30.1589	32.2753	335.9940
LNC_000057	15.5034	232.4060	29.6239	28.7508	306.2843
LNC_000040	17.5395	215.5778	28.0434	30.6050	291.7658
ENSMUST00000152365.1	14.3560	227.8362	26.2226	22.5889	291.0038
ENSMUST00000137810.2	13.5911	218.9375	25.9034	24.7821	283.2142
LNC_000279	12.8857	217.3779	26.5701	26.3653	283.1992
LNC_000353	15.4114	207.9657	27.1850	29.2075	279.7697
ENSMUST00000154414.1	12.3479	211.8834	24.6985	27.0541	275.9841

*BP*, biological process; *CC*, cellular component; *MF*, molecular function.

### C value is superior to log2 FC and p-value in miRNA operation results

**Figure 1:**
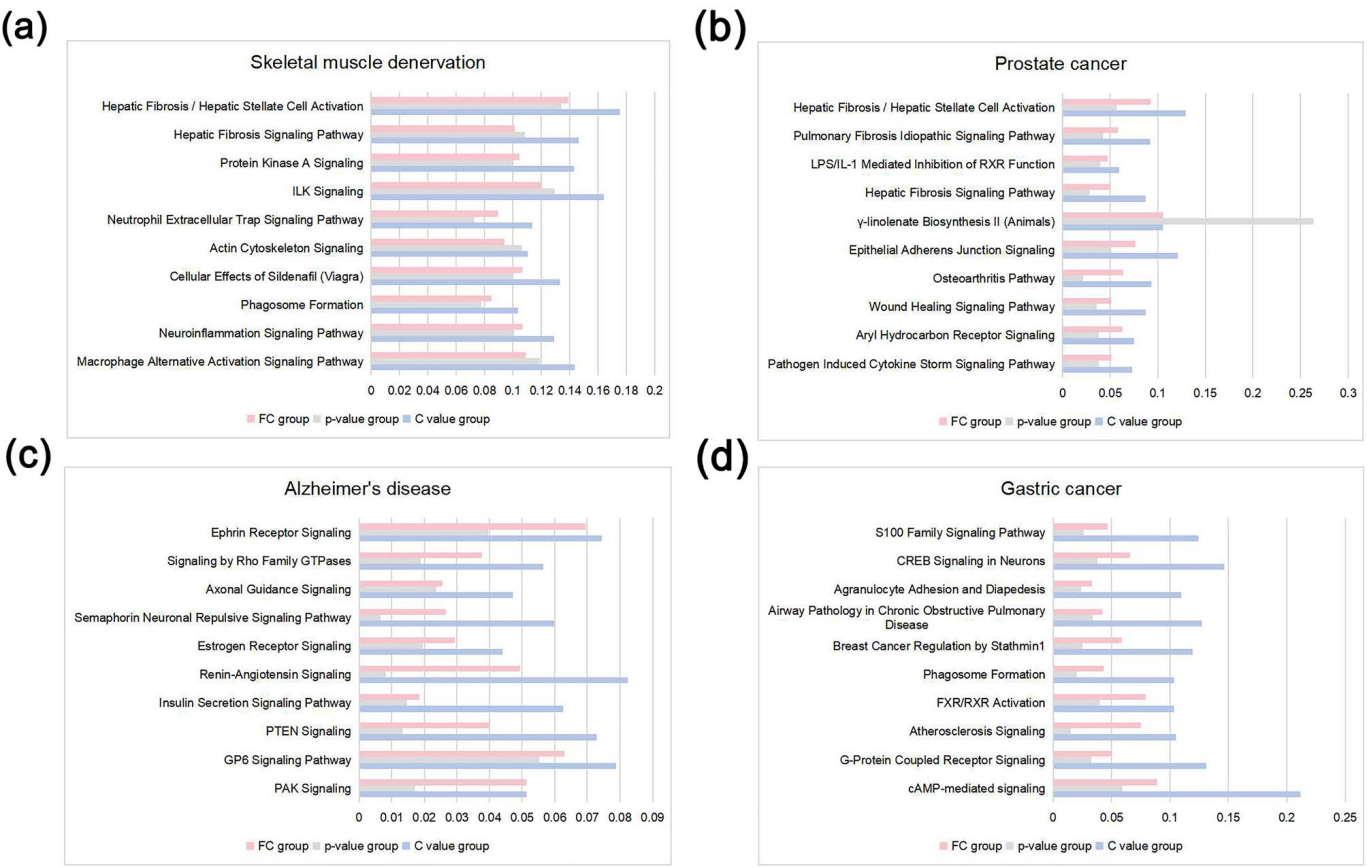
Proportion of three groups in each IPA canonical pathway (**a**) Skeletal muscle denervation. (**b**) Prostate cancer. (**c**) Alzheimer's disease. (**d**) Gastric cancer. (FC group: the collection of the top10 FC miRNAs’ predictive target mRNAs; p-value group: the collection of the top10 p-value miRNAs’ predictive target mRNAs; C value group: the collection of the top10 C value miRNAs’ predictive target mRNAs). Picture drawn by Microsoft Excel.

**Figure 2:**
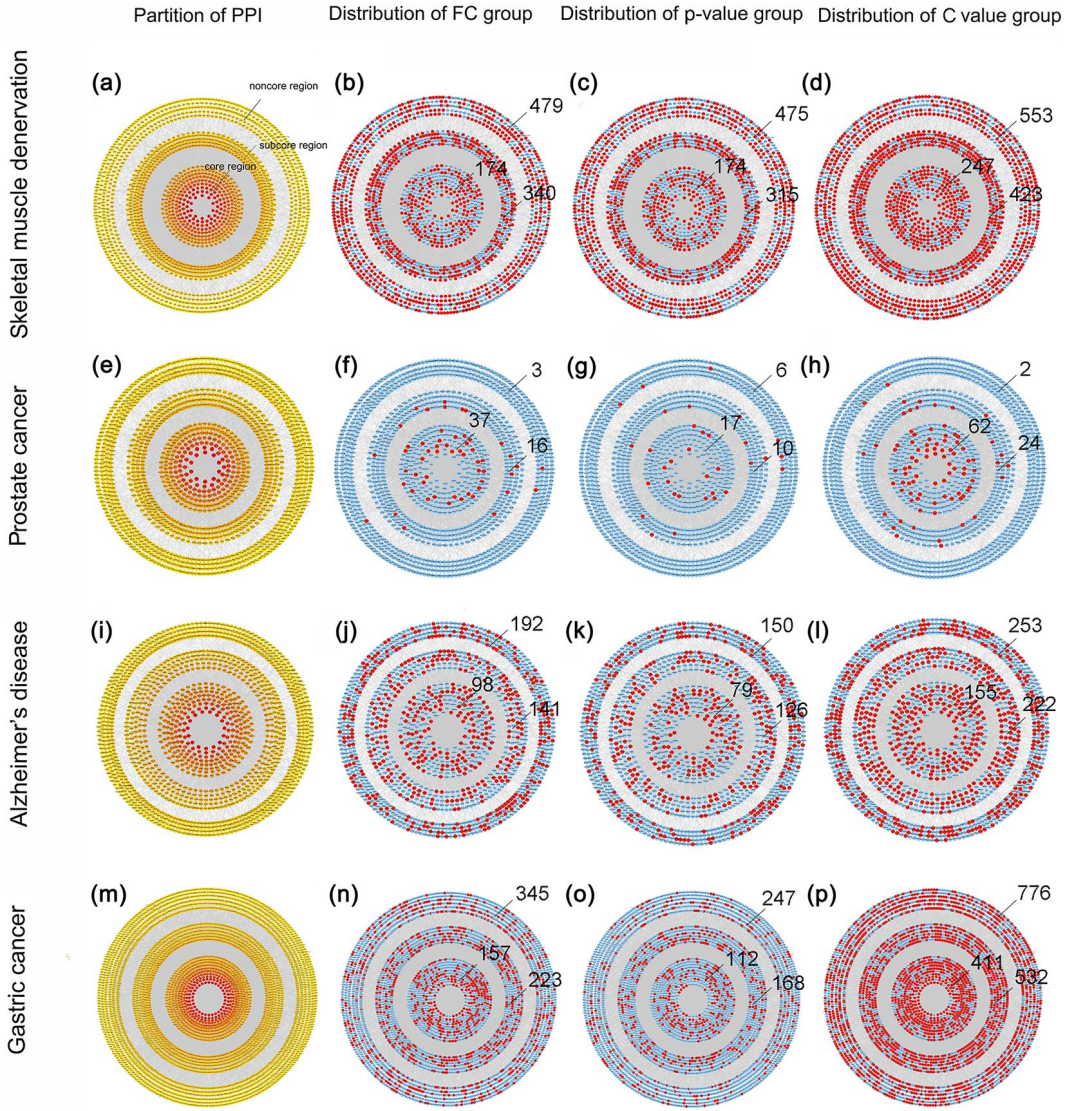
Partition of PPI network and distribution of each group in PPI network. (**a**,**e**,**i**,**m**) PPI network of DEGs in the Skeletal muscle denervation dataset, Prostate cancer dataset, Alzheimer's disease dataset and Gastric cancer dataset. The degree of each node was calculated. The larger the degree of the node, the darker the color and the closer the position is to the center. The top 20% nodes are defined as core regions, the top 20%-50% nodes are defined as sub core regions, and the remaining nodes are noncore regions. (**b**,**f**,**j**,**n**) Distribution of FC group in PPI network. (**c**,**g**,**k**,**o**) Distribution of p-value group in PPI network. (**d**,**h**,**l**,**p**) Distribution of C value group in PPI network. Red is the selected node, blue is the unselected. Number of genes in core region, sub core region and noncore region of each group has been tagged. STRING v11.0 was used to generate protein interactions, and the resulting network was visualized using Cytoscape v3.7.2. (FC group: the collection of the top10 FC miRNAs’ predictive target mRNAs; p-value group: the collection of the top10 p-value miRNAs’ predictive target mRNAs; C value group: the collection of the top10 C value miRNAs’ predictive target mRNAs).

**Figure 3:**
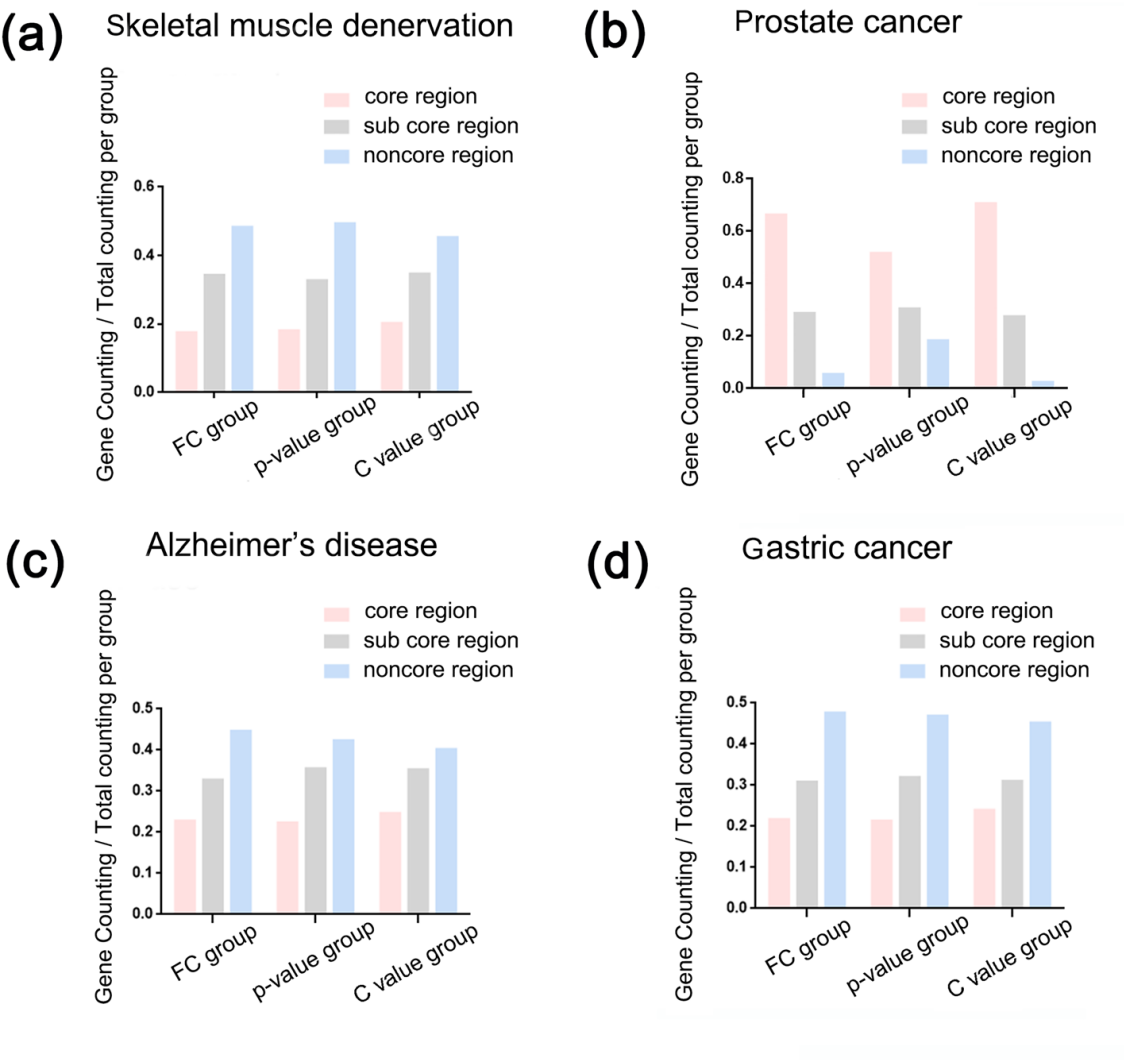
Statistics on the distribution of each group in the PPI network. (**a**) Skeletal muscle denervation. (**b**) Prostate cancer. (**c**) Alzheimer's disease. (**d**) Gastric cancer. The ratio of the number of genes in each group in different regions.

In each data set, the most significant enriched IPA canonical pathways were obtained by core analysis (Supplementary Table 7). We took the intersections of DEGs with the predicted target genes of top10 C value miRNAs, top10 FC miRNAs and top10 p-value miRNAs respectively, and then calculated the proportion of these intersections in the above pathways. It was found that the proportion of top10 C value miRNAs target genes was significantly larger than that of top10 FC miRNAs, top10 p-value miRNAs in most pathways (Fig. 1). We built several PPI networks based on DEGs, and calculated the degree of each node. The node with a larger degree had a darker color and was closer to the center. Then we divided these nodes into the core region (top 20% of degree), sub core region (top 20%-50% of degree) and noncore region (bottom 50% of degree) (Fig. 2a,e,i,m). In the PPI network, the predicted target genes of top10 C value miRNAs, top10 FC miRNAs and top10 p-value miRNAs were labeled in red (Fig. 2). It was found that the number of top10 C value miRNAs’ target genes in each region were larger than those of top10 FC miRNAs, and top10 p-value miRNAs, and the C value group are more concentrated in core region (Fig. 3) (Table 4).

**Table 4:** The ratio of the number of genes in each group in different regions.

	FC group			p-value group			C value group		
	Core region	Sub core region	Noncore region	Core region	Sub core region	Noncore region	Core region	Sub core region	Noncore region
Skeletal muscle denervation	0.1752	0.3424	0.4824	0.1805	0.3268	0.4927	0.2020	0.3459	0.4522
Prostate cancer	0.6607	0.2857	0.0536	0.5152	0.3030	0.1818	0.7045	0.2727	0.0227
Alzheimer's disease	0.2274	0.3271	0.4455	0.2225	0.3549	0.4225	0.2460	0.3524	0.4016
Gastric cancer	0.2166	0.3076	0.4759	0.2125	0.3188	0.4687	0.2391	0.3095	0.4514

**Figure 4:**
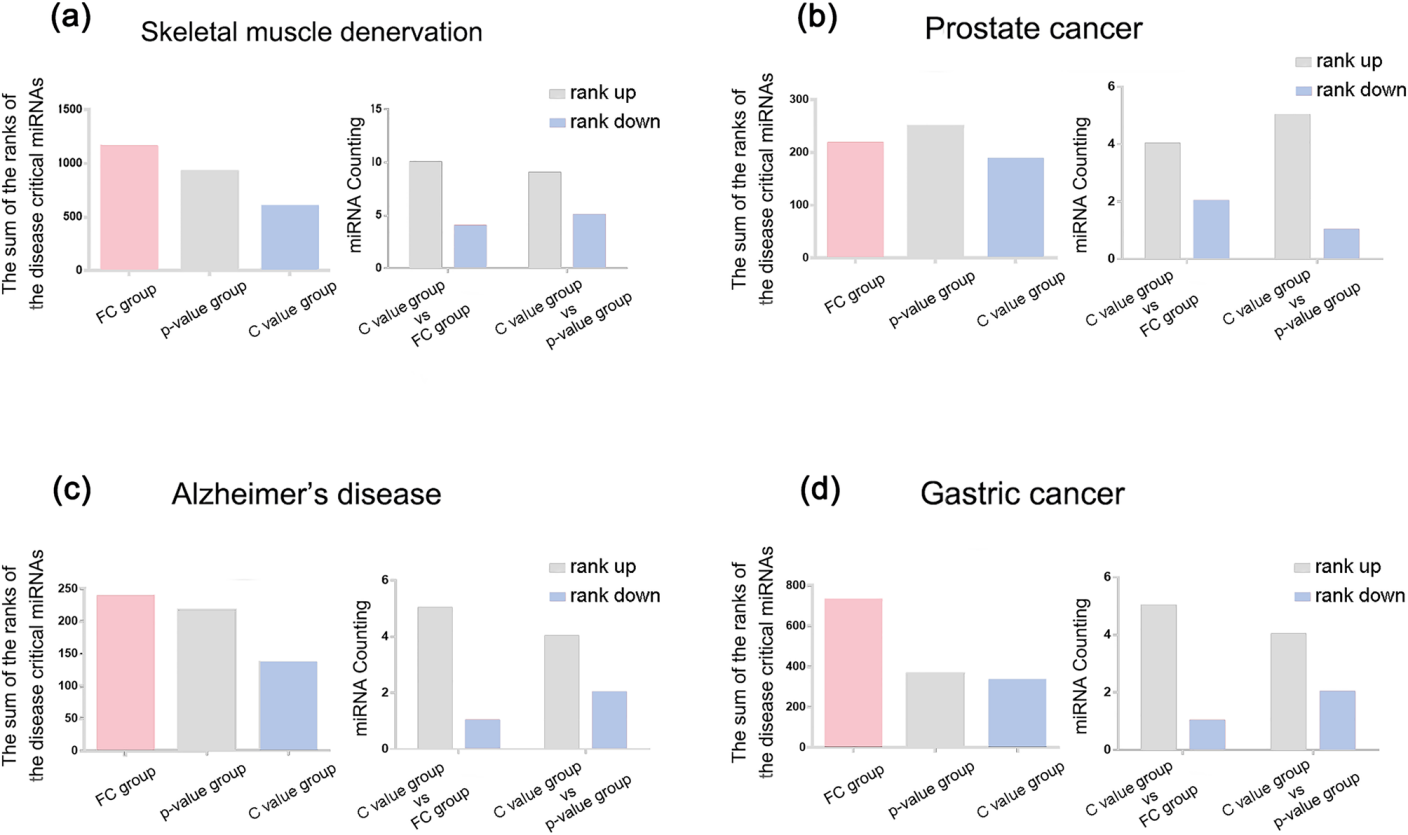
After sorting with C value, the ranking of disease critical miRNAs increased integrally. (**a**) Skeletal muscle denervation. (**b**) Alzheimer's disease. (**c**) Prostate cancer. (**d**) Gastric cancer. Left: the sum of the ranks of disease critical miRNAs by the three indexes. Right: The number of mRNAs that rank up or down. (FC group: the collection of the top10 FC miRNAs’ predictive target mRNAs; p-value group: the collection of the top10 p-value miRNAs’ predictive target mRNAs; C value group: the collection of the top10 C value miRNAs’ predictive target mRNAs).

Based on extensive literature, we identified 14 skeletal muscle growth regulatory miRNAs, 6 Alzheimer’s disease associated miRNAs, 6 prostate cancer associated miRNAs, and 6 gastric cancer associated miRNAs and found that when DE miRNAs were sorted by C value, the sum of the ranks of these miRNAs was significantly smaller than that of the other two indexes, which means that these miRNAs sequences increased integrally (Fig. 4). When sorting by C value versus sorting by absolute Log2 FC/ p-value, most of the disease critical miRNAs ranked up (Fig. 4) (Supplementary Table 8).

### C value is superior to log2 FC and p-value in lncRNA operation results

**Figure 5:**
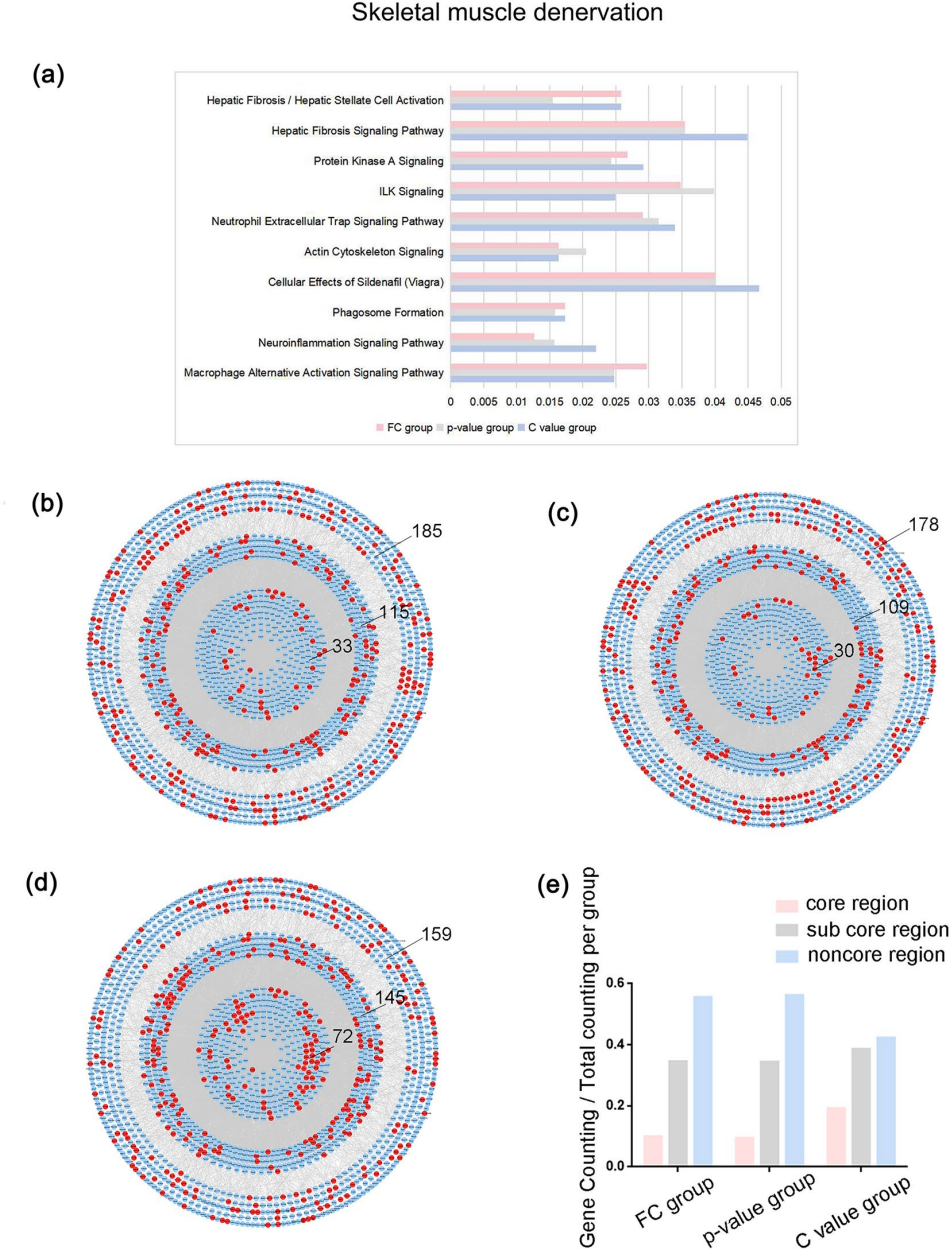
LncRNA operation results for skeletal muscle denervation data set (**a**) The ratio of predicted target genes to the total genes in IPA canonical pathways. The distribution of (**b**) top10 FC, (**c**) top10 p-value and (**d**) top10 C value lncRNAs’ predictive target mRNAs in the PPI network. Number of genes in core region, sub core region and noncore region of each group was tagged. (**e**) The ratio of the number of genes in each group in different regions. (FC group: the collection of the top10 FC lncRNAs’ predictive target mRNAs; p-value group: the collection of the top10 p-value lncRNAs’ predictive target mRNAs; C value group: the collection of the top10 C value lncRNAs’ predictive target mRNAs).

In the skeletal muscle denervation data set, we calculated the proportion of the predicted target genes of top10 C value lncRNAs, top10 FC lncRNAs, and top10 p-value lncRNAs in the most enriched IPA canonical pathways respectively, and found that the proportion of the genes regulated by top10 C value lncRNAs was larger than that of top10 FC lncRNAs and top10 p-value lncRNAs (Fig. 5a). It was found that the number of top10 C value lncRNAs’ target genes in each region were larger than those of top10 FC lncRNAs, and top10 p-value lncRNAs and the C value group are more concentrated in the core region (Fig. 5b–e) (Table 5).

**Table 5:** The ratio of the number of genes in each group in different regions.

	FC group			p-value group			C value group		
	Core region	Sub core region	Noncore region	Core region	Sub core region	Noncore region	Core region	Sub core region	Noncore region
Skeletal muscle denervation	0.0990	0.3453	0.5555	0.0946	0.3438	0.5615	0.1915	0.3856	0.4229
Adipocyte differentiation	0.2727	0.3636	0.3636	0.3000	0.3000	0.4000	0.4800	0.3600	0.1600

**Figure 6:**
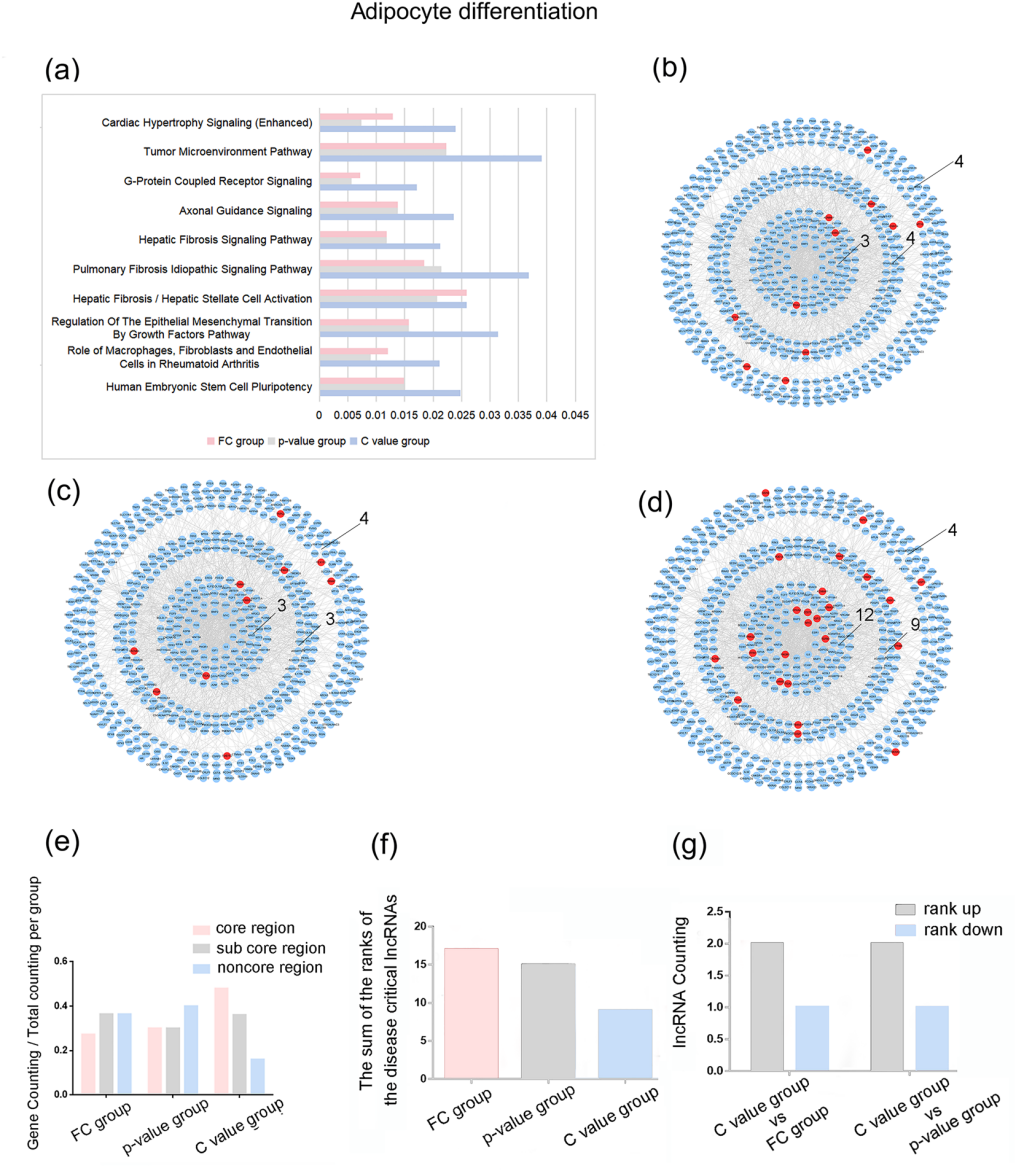
LncRNA operation results for adipocyte differentiation data set (**a**) The ratio of predicted target genes to the total genes in IPA canonical pathways. The distribution of (**b**) top10 FC, (**c**) top10 p-value and (**d**) top10 C value lncRNAs’ predictive target mRNAs in the PPI network. Number of genes in core region, sub core region and noncore region of each group was tagged. (**e**) The ratio of the number of genes in each group in different regions. (**f**) The sum of the ranks of adipocyte differentiation associated lncRNAs by the three indexes. (**g**) The number of adipocyte differentiation associated lncRNAs that rank up or down. (FC group: the collection of the top5 FC lncRNAs’ predictive target mRNAs; p-value group: the collection of the top5 p-value lncRNAs’ predictive target mRNAs; C value group: the collection of the top5 C value lncRNAs’ predictive target mRNAs).

Since there are relatively few DE lncRNAs and DE mRNAs in the adipocyte differentiation data set, we take top5 C value lncRNAs, top5 FC lncRNAs, top5 p-value lncRNAs. The proportion of the genes regulated by top5 C value lncRNAs was larger than that of top5 FC lncRNAs and top5 p-value lncRNAs in enriched IPA canonical pathways (Fig. 6a). It was found that the number of top5 C value lncRNAs’ target genes in each region were larger than those of top5 FC lncRNAs, and top5 p-value lncRNAs and the C value group are more concentrated in the core region (Fig. 6b–e) (Table 5). And when DE lncRNAs were sorted by C value, the adipocyte differentiation associated lncRNAs sequences increased integrally than that of the other two indexes (Fig. 6f–g) (Supplementary Table 8).

### Efficiency comparison of different ncRNAs

Firstly, the results of IPA canonical pathways were analyzed, and the proportion of the C value group in the top10 pathways was calculated compared with the other two groups. We found that in miRNA data set, the efficiency of the C value group was improved by 61% compared with the FC group, and by 145% compared with the p-value group. In lncRNA data set, the C value group increased by 39% compared with the FC group, and by 78% compared with the p-value group (Table 6). Then, by analyzing the results of PPI network and calculating the ratio of the C value group in core region compared with the other two groups, we found that the C value group in miRNA data set increased by 10% compared with the FC group and by 18% compared with the p-value group. In lncRNA data set, the C value group increased by 85% compared with the FC group, and by 81% compared with the p-value group. In general, there is little difference between the results of miRNA and lncRNA, and a greater difference occurs between different data sets, which may be related to the quality of data sets (Table 7).

**Table 6:** Efficiency comparison of C value in IPA canonical pathways.

ncRNA	Dataset	Increase rate (C value vs. FC)	The average of the increase rate (C value vs. FC)	Increase rate (C value vs. p-value)	The average of the increase rate (C value vs. p-value)	
microRNA	Skeletal muscle denervation	0.29	0.61	0.30		1.45
	Prostate cancer	0.40		0.50		
	Alzheimer's disease	0.53		1.91		
	Gastric cancer	1.20		3.09		
lncRNA	Skeletal muscle denervation	0.07	0.39	0.09		0.78
	Adipocyte differentiation	0.71		0.86

**Table 7:** Efficiency comparison of C value in PPI network.

ncRNA	Dataset	Increase rate of core region ratio (C value vs. FC)	The average of the increase rate (C value vs. FC)	Increase rate of core region ratio (C value vs. p-value)	The average of the increase rate (C value vs. p-value)	
microRNA	Skeletal muscle denervation	0.15	0.10	0.12		0.18
	Prostate cancer	0.07		0.37		
	Alzheimer's disease	0.08		0.11		
	Gastric cancer	0.10		0.13		
lncRNA	Skeletal muscle denervation	0.93	0.85	1.02		0.81
	Adipocyte differentiation	0.76		0.60		

## Discussion

After high-throughput sequencing, it is common to screen ncRNA according to expression differences. But this may lose a lot of valuable information and lead to biased results. Considering the strong regulatory function of ncRNA on gene expression, there is currently no indicator to characterize the regulatory function and participation degree of ncRNA on transcriptome expression to help us evaluate and screen ncRNA. Here we designed a new algorithm PDNT to calculate the Contribution value, which is defined as a quantitative indicator of the participation degree of ncRNA in transcriptome.

To test the superiority of C value, we compared it with absolute Log2 FC and p-value. Log 2 FC reflects the expression change of ncRNAs and p-value reflects how significant the change is. The two indexes of each DE RNA were obtained after the traditional whole transcriptome sequencing, and many follow-up studies have partially referenced Log2 FC and p-values in selecting the target gene^9,10,16^. We analyzed four microRNA data sets and two lncRNA data sets, and compared the C value with Log2 FC and p-value in each data set. First, we performed enrichment analysis on DEGs to obtain the most enriched IPA canonical pathways. We found that top C value ncRNAs targeted more genes in these pathways than FC and p-value groups, which may suggest that top C value ncRNAs have greater regulatory potential for enriched pathways. Further, we constructed a PPI network based on DEGs, partitioned the PPI by degree, and then observed the distribution of the three groups in different partitions. It was found that the number of target genes of top C value ncRNAs in each region was greater than that of the other two groups. At the same time, a larger proportion of target genes in the C value group were concentrated in the central region of the PPI. It suggests that the top C value ncRNA has a broader and more important influence on the PPI network than the other two groups. Finally, based on literature search, we obtained key ncRNAs that regulate various pathological/ physiological processes, and then tested the screening effect of the three indicators on these key ncRNAs in the datasets. It was found that using the C value to rank ncRNAs made the overall ranking of these key ncRNAs higher than the other two indicators. This suggests that ncRNAs screened with C values have a greater potential for regulating pathological/physiological processes.

In order to correct the bias caused by only considering expression differences when screening ncRNA, many analysis methods and databases have been derived, such as GSEA^11^ IPA^12^, David^13^, Catmap^14^ and GlobalTest^15^. Their analytical methods have different priorities, but the general idea is the same, that is, to perform functional annotation on the RNA profile. But through these methods, we can only observe which genes and pathways are associated with ncRNAs. We do not have a measure to evaluate the participation degree of ncRNA in transcriptome. This lack may result in our inability to assess the priority of two ncRNAs when their target genes are close in number. Or when the two ncRNA regulate similar pathways, we cannot judge their participation degree in the expression regulation of the transcriptome. The algorithm PDNT proposed in this study is based on these pathway analysis methods. We hope to make better use of the pathway enrichment results to evaluate ncRNA and we integrated more valuable information to optimize the screening efficiency of ncRNA. The limitation of this study is that we only calculated based on one pathway enrichment method. In the subsequent study, we will compare the differences between the results calculated based on different pathway enrichment methods, to provide more inspiration and help for related research.

Based on the above evidence, the PDNT is an efficient algorithm for calculating the participation degree of ncRNA in transcriptome based on pathway analysis. We found that the PDNT algorithm provides a measure from another view compared with the log2FC and p-value and it may provide more clues to effectively evaluate ncRNA.

## Methods

### Prediction of ncRNAs’ target mRNAs

MiRNA: MiRNAs target genes prediction software, miRanda-3.3a (http://www.microrna.org/) ^22^, uses a weighted dynamic programming algorithm to calculate the optimal sequence complementarity between a mature microRNA and a given mRNA. The main parameters are: -sc 140, -en -10, -scale 4, -strict -out.

LncRNA: The target genes of lncRNAs are predicted by co-expression analysis among samples. The Weighted Gene Correlation Network Analysis (http://www.r-project.org/) ^23^ was used to calculate Pearson correlation coefficients. The absolute value of the Pearson correlation coefficient ≥ 0.90, p-value < 0.01 and FDR < 0.01 was saved.

### GO and KEGG pathway enrichment analysis

In this study, the screening criteria for DEG were p < 0.05 and absolute Log2 FC ≥ 1.

GO is a database established by Gene Ontology consortium (http://www.geneontology.org), which includes three parts: molecular function, biological process and cell composition. KEGG is based on the Kyoto Encyclopedia of Genes and Genomes (KEGG) database (http://www.genome.ad.jp/kegg/), Fisher exact test and × 2 test were used. Enrichment analysis of differentially expressed genes was performed using clusterProfiler R software package^24^, and gene length bias was corrected. The corrected p-value less than 0.05 was considered to be significantly enriched by differentially expressed genes.

### C value mathematical model and its calculation

**Figure 7:**
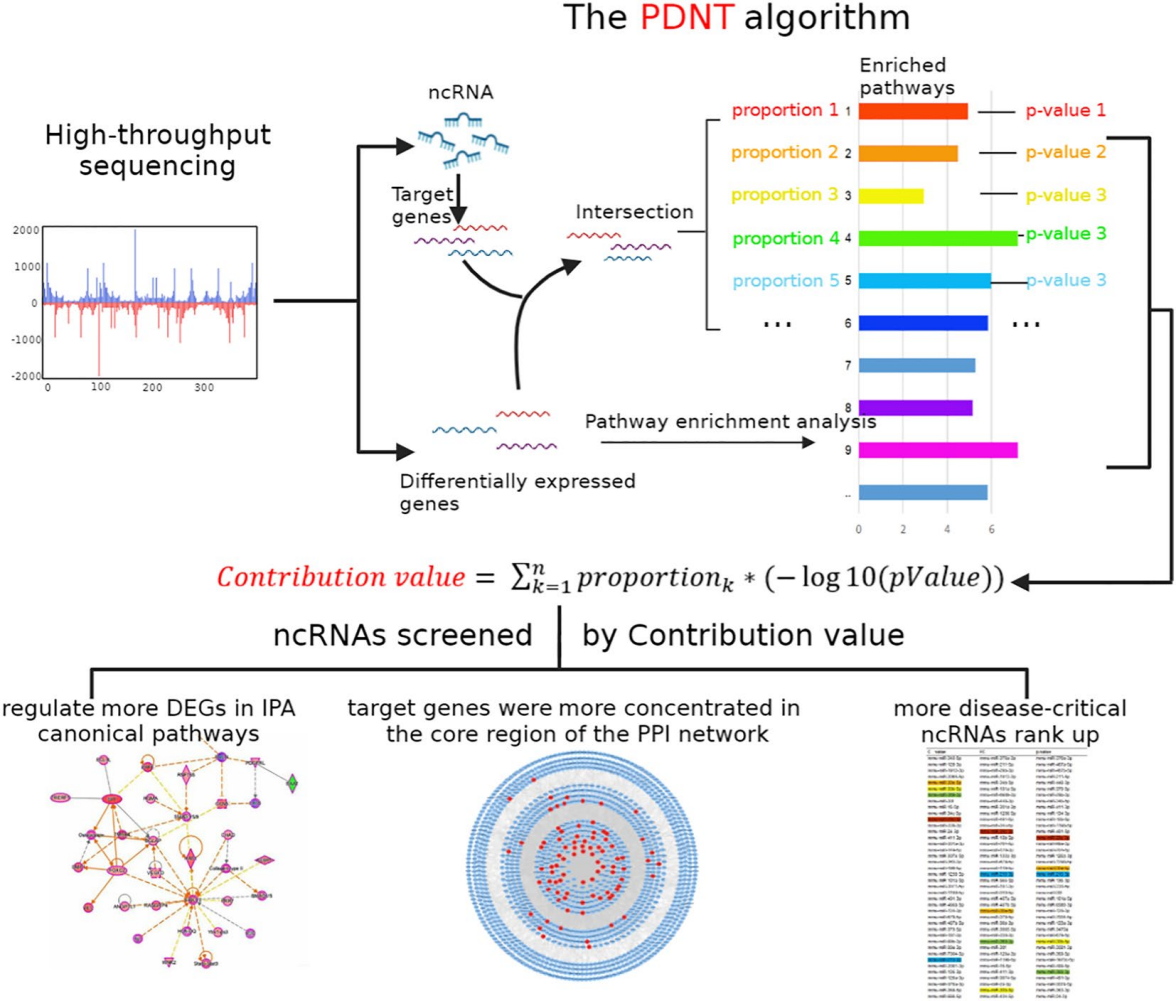
The operation and verification process of the PDNT algorithm.

The C value of each DE ncRNA is calculated using the PDNT algorithm (Fig. 7):

$$C value= \sum_{k=1}^{n}{Proportion}_{k}*(-\mathrm{log}10(pValue)$$

p-value is the p-value of the pathway enriched by DEGs; Proportion refers to the proportion of the intersection between ncRNA target genes and DEGs in each pathway; n represents the number of pathways enriched by DEGs.

### Ingenuity pathway analysis (IPA) core analysis

IPA core analysis of DEGs (p < 0.05 and absolute Log2 FC ≥ 1) was performed using IPA (version 81,348,237, Qiagen), showing top10 canonical pathways according to p-value.

### PPI network for DEGs

For each dataset, the STRING v.11.0 database was used to construct the PPI network based on DEGs. The images were then drawn by cytoscape3.72 (San Diego, CA, USA).

### Retrieval and statistics of key miRNAs and lncRNAs

We searched PubMed (http://www.ncbi.nlm.nih.gov/pubmed) for miRNAs that play important roles in skeletal muscle denervation, Alzheimer's disease, prostate cancer and gastric cancer, respectively. The key words were "skeletal muscle AND microRNA", "Alzheimer's disease AND microRNA", "prostate cancer AND microRNA", and "gastric cancer AND microRNA". Next, we retrieved the lncRNAs that play an important role in skeletal muscle denervation and adipocyte differentiation. Keywords: "skeletal muscle AND lncRNA" and "adipocyte differentiation AND lncRNA". The results were shown in Table 8.

**Table 8:** The key miRNAs and lncRNAs.

	MicroRNA/LncRNA
Skeletal muscle denervation	miR-204-5p^25^, miR-214^26^, miR-10b-5p^27^, miR-152^28^, miR-27a^29^, miR-18a^30^, miR-139-5p^31^, miR-159/497^32^, miR-29c^33^, miR-34b^34^, miR-22^35^, miR-34c^36^, miR-378a-3p^37^, miR-206^38^
Prostate cancer	miR-20a, miR-20b, miR-23b, let-7a^18^, miR-155-5p^39^, miR-218-5p^40^
Alzheimer's disease	miR-30b^41^, miR-29c^42^, miR-369-3p, miR-369-5p^43^, miR-30e, miR-210^44^
Gastric cancer	miR-148a^45^, miR-20a^46^, miR-181b^47^, miR-143^48^, miR-218^49^, miR-17^50^
Adipocyte differentiation	MIAT, LINC02202, LINC01119^20^

### Data Analysis

The analysis platform is R 3.6.1 and the R package is clusterProfiler. The database is org.Mm.eg.db developed with the R package.

## Supplementary Information

Supplementary Information.

## Data Availability

All data generated or analysed during this study are included in these published articles [and their supplementary information files]^16–20^.

## Abbreviations

FC: Fold change
GSEA: Gene set enrichment analysis
IPA: Ingenuity pathway analysis
C value: Contribution value
DEG: Differentially expressed gene
PPI: Protein–protein interaction
ncRNA: Non-coding RNA
miRNA: MicroRNA
APP: Amyloid beta precursor protein
GO: Gene Ontology
KEGG: Kyoto encyclopedia of genes and genomes
BP: Biological process
CC: Cellular component
MF: Molecular function
